# Financial support failure and health results: The Peruvian case

**DOI:** 10.1371/journal.pone.0277327

**Published:** 2023-02-03

**Authors:** Jorge Guillen

**Affiliations:** ESAN University, Lima, Peru; University of Seville, SPAIN

## Abstract

The paper shed the light in analyzing the efficiency of Peruvian Government Financial Support in comparison with some countries in the Latin American Region and worldwide. The Covid 19 Pandemic enforced governments to apply the “Hammer Blow” which affected negatively the economy producing recession and unemployment. Governments offset the latter effect by applying subsidy policies to the Poor and then reduce the negative economic consequences of the general lockdown without getting COVID. Our study performs a Difference and Difference Model (DID) to evaluate the effectiveness of the latter policy.

## 1. Introduction

Many governments worldwide have made the decision to assume the opportunity cost of compulsory social isolation of the poor. In particular, the Peruvian president Martin Vizcarra declared a State of National Health Emergency due to the Covid-19 pandemic. The objective of this rule was to ensure that the majority of the vulnerable population remains in their homes isolated from all contagion. Along this line, the Peruvian government has chosen to apply a policy of granting monetary subsidies progressively to mitigate the adverse economic impact of compulsory social isolation. The four types of bonds cover 6.1 million households for an amount of S/ 4.46 billion [1]. Also, a series of measures have been initiated to alleviate the effect of isolation on SMEs and large companies through programs such as REACTIVA Peru. The latter program subsidy for firms may avoid layoffs and offset fixed labor cost deteriorated by the economic shutdown [see 2, 3].

The main objective of the paper is to find the efficiency of the Financial Support to the poor during the State of National Health Emergency. [3] also analyzed economic consequences of the lockdown or “hammer blow” which attempted to reduce Covid 19 cases and permitted health assistance to most of the population in Intensive Care Unit (ICU). Italy and USA had peaks of 812 and 2,349 deaths during the first wave of cases (March- June 2020). Emerging Countries like Peru, Brasil and Chile, the number hit 584, 650 and 96 death cases during the same period [4]. There are more case waves with significant effect on death. Lately, we do not have significant amount of deaths since vaccination has progressed worldwide. The data of our study span the first wave of Covid 19 cases

In addition, [3] shows heterogeneous economic and health results after the application of financial support for the Poor. Emerging Economies had more negative consequences in comparison with Developed Economies. The different infrastructure, degrees of informality and ability to cope between the latter set of countries induced heterogeneous results. Our study complements the results of Loayza. Also in [5] there is a discussion of low fiscal space characterized by emerging economies.

[6] show how stimulus payments to low-income households increased consumer spending sharply, but little of this increased spending flowed to businesses most affected by the COVID-19 shock, dampening its impacts on employment. [7] shows for a sample in UK that the effect of the Pandemic was homogeneous across gender However for [8] the effect of COVID on employment was asymmetrical and deepened by age for his sample in the US. Our study focusses the initial Pandemic effects in Peru, an emerging economy that may have different effects according to [3] and we will focus the attention on the financial support for vulnerable families in order to offset economic and health negative effects within latter groups.

Next section presents the subsidy policy during the Pandemic for a set of countries in the Latin American Region as well as some Developed Countries. Some Statistics are presented which show preliminary results in lines with our hypothesis set up above. Then a Model of Difference and Difference (DID) is presented along the results, to finally conclude.

## 2. Case study of some countries providing subsidies during pandemic

Next section shows Case Studies providing subsidies to the poor during Pandemic. We cover Europe, US and some Latin American countries. Then we can make a comparative analysis in order to achieve a preliminary result that will be complemented with the empirical section.

### 2.1 The Peruvian case

Since August 2020, Peruvian Government provided demand stimulus given by the following programs:

**Table:** 

• Bond “Yo me quedo en casa”
• Bond “Rural”
• Bond Familiar Universal
• Bond Independiente • Bond “Mype”

The Bond "Yo me quedo en casa" is aimed at Households in poverty or extreme poverty according to the Household Targeting System (SISFOH) and is granted under the responsibility of the Ministry of Development and Social Inclusion [9].

The Bond “Rural” aimed Households in conditions of poverty or extreme poverty in rural areas according to the Household Targeting System (SISFOH). For details see the Emergency Decrees No. 027–2020 and No. 033–2020 [10].

The Universal Family Bond is targeted for households whose members are not registered in the Application for the Centralized Registry of Payrolls and Public Sector Data (AIRHSP) or in the available private payroll, and who have not received or will not receive the monetary subsidy from the “Independent” Bond, “Yo me quedo en Casa” and “Rural” Bond.

Finally, the “Mype” Perfect Suspension Bond is for workers who are in a perfect suspension of job. It is not a laidoff situation, is just a suspension of payroll payments. They were mostly for people who belong to the microenterprise labor regime and whose gross remuneration is up to US$ 700 dollars per month.

More than 6 million people were benefited from the first provision of Bonds which were set in the first half of 2020. The idea is to offset drop in consumption of the vulnerable population within the country.

### 2.2 The case of US and Europe

Not only Latin America has granted the subsidy to vulnerable households. US and Europe has also perform subsidies in a greater proportion in comparison to the countries of the Latin American Region [11]. Table 1 below summarizes the amounts granted in the northern hemisphere

**Table 1:** Monetary subsidy in US and Europe.

**Monetary Subsidy–USA—2020 (US$)**				
USA	Stimulus Checks	1,200.00 dollars		
	Bond per family	2,400.00 dollars		
	Bond for under 16 years	500.00 dollars		
	**Average**	**1,366.67 dollars**		
**Monetary Subsidy—Europe—2020 (US$)**				
**Country**	**Social Program**	**Amount**		
		Country Currencys		Dollars
Spain	Bond for temporary workers	430.00	euros	490.20
Italy	Bond for independent workers	600.00	euros	684.00
France	Bono for Kids	250.00	euros	285.00

Source: Central Banks of each country [12]

Source: Press and Central Bank of each country [13]

Elaboration: Own

Spain, among various aids to compensate expenses of vulnerable families, has granted a subsidy of 430 euros for temporary workers inactive for two months. The United States has provided subsidies of $1,200, $2,400 for couples and $500 for those under 16 years of age. The next figure below shows a comparative analysys that shows a preliminary resulto of a lack of stimulus in Peru. Next section presents the Difference and Difference Model (DID) to test the cost-benefit of the financial support to the peruvian por during the first phase of the pandemic.

### 2.3 Chilean case

Chile has provided to its vulnerable population 275,000 pesos chilenos, equivalent to US$330. The stipend is granted to formal and informal families for three times since the beginning of the pandemic until august 2020.

Through the Bono Covid portal (https://www.bonocovid.cl/) a vulnerable person can realize if it is possible to access for the social aid during the Pandemic [14]. Like the countries in the region, the Subsidy Bond aims to support the most vulnerable families

People who qualify for the subsidy may apply for:

- People with Family Subsidy (SUF). In this case, the SUF must have been in effect as of February 29. They will receive $50,000 Chilean pesos (US$62.93) for each head of family entitled to Family Subsidy.
- Families of the Securities and Opportunities Subsystem. These people must have been incorporated into that subsystem on February 29. The amount of $50,000 (US$62.93 dollars) per family will be awarded.
- Households of the 60% most vulnerable, according to the Social Registry of Households (RSH). People without formal income from work or pension, and without benefits such as the Family Allowance. These homes must have been in that condition as of April 1. The amount of $50,000 (US$62.93 dollars) per household will be awarded.

**Fig 1:**
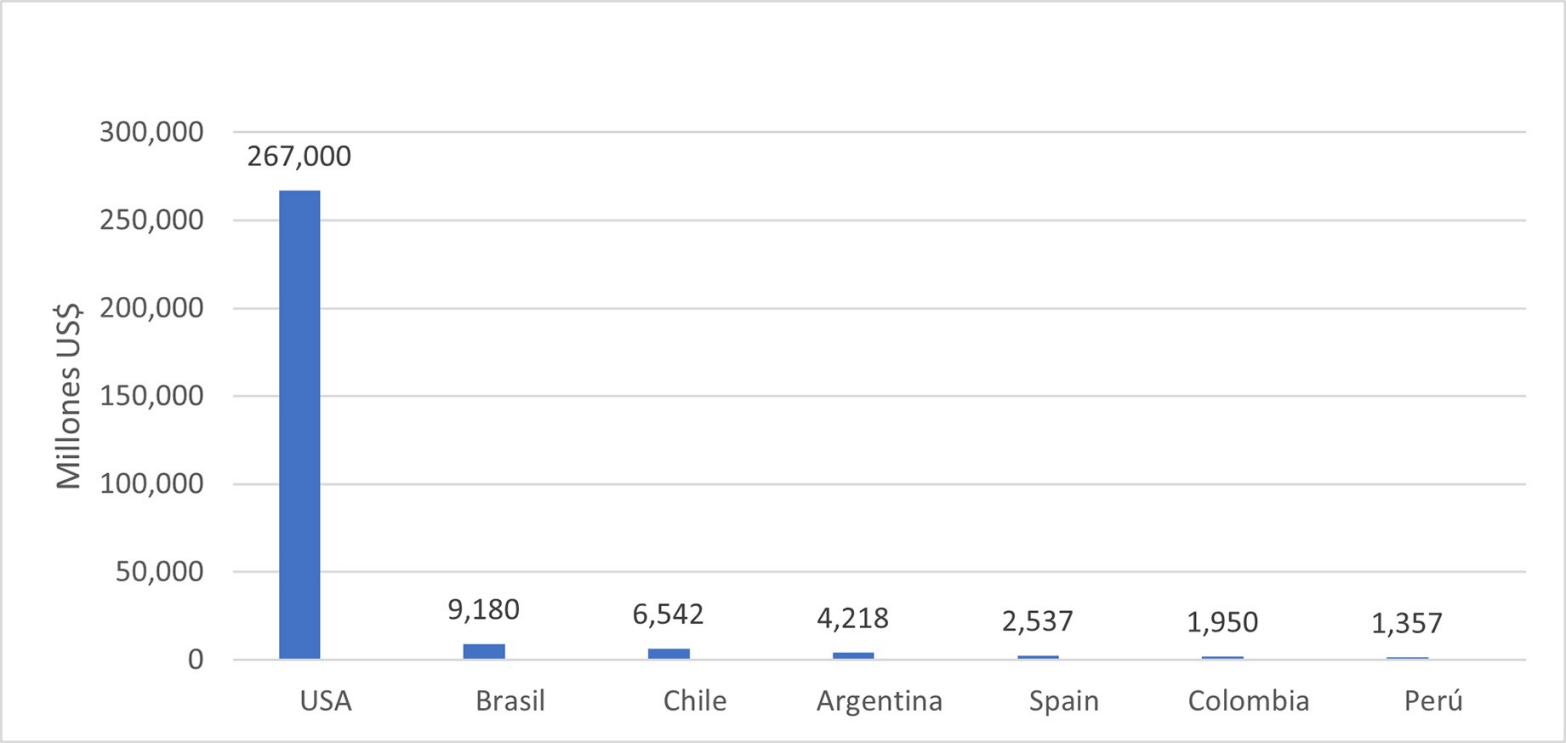
COVID monetary subsidty (US$). **Source:** Central Banks [15]. *Average of the subsidy for the first six months.

Attempts have been made to reach the formal and informal middle classes, whose income has deteriorated by 30%. In Peru the scope only hit the vulnerable. Here they attempt to reach low middle class. As can be seen from Fig 1 above, the amounts and coverage are significant. It is intended to grant here in tranches up to 500,000 pesos (equivalent to US$600 dollars)

If a person belongs to more than one group, the bonuses are not added and only one payment is considered for the family. All this framework is within the "Chile Attends" program. Bonds in Chile have been delivered to different subgroups of the population, very similar to the Peruvian case.

### 2.4 Social programs in Chile (see [16])

As mentioned above, the people who rate Chile’s Covid 19 Bonds are three groups. Let’s define in detail who integrates each of the mentioned groups:

The first group is the Single Family Subsidy (SUF), intended for low-income people who cannot access the Family or Maternal Allowance benefit, because they are not workers affiliated with a pension system. The subsidy is 13,155 Chilean pesos (US$16.56 American dollars). The disabled are given double.

People who are entitled to receive the Family Subsidy can access free medical benefits. In the case of pregnant women, they can request the Maternal Subsidy from the fifth month of gestation, from which they stop receiving the Family Subsidy. This subsidy covers the entire pregnancy and is paid through the Social Security Institute (IPS).

In the other group of candidates to receive the Covid-19 bonus from Chile is: The Chilean Securities and Opportunities Subsystem, which is a non-applicable benefit focused on providing comprehensive and continuous support to the most vulnerable individuals and families, so that they through their own effort and commitment overcome their condition of extreme poverty. They are people who are not affiliated with a pension system either.

This program is subdivided into:

- Family Program: which is aimed at individuals and families who are in a situation of extreme poverty and vulnerability.
- Links Program: a program for people over 65 years of age, which seeks to provide resources to improve the living standards of the elderly.
- Calle Program: program aimed at adults to enable their social inclusion. Psychosocial services are also provided.
- In the case of children and adolescents, it makes it possible for minors to get out of the street situation and they act through programs with psychosocial, family and community intervention.
- Opening Paths Program: program aimed at children, young people and their families who have an adult in a penitentiary center.

In Chile there are seven sections of socioeconomic classification and they go from section 1 to the seventh. Being the first, the one with the lowest income. The most vulnerable section of the population that also receives social aid would be found in the first socioeconomic section.

### 2.5 Bolivian case (See [17–19])

In Bolivia, an average of 466 bolivianos (US$70) has been distributed through three deliveries. In total, the Bolivian government has allocated US$ 500 million dollars in bonds for its low income population. These are somewhat low amounts considering that the average of the Latin American region has three times more than that equivalent amount in US dollars (See Fig 1 above).

The "Universal Bonus" is granted to any person who is included in the economically active population (PEA). That is, the population that fluctuates between 18 and 60 years of age and who have not contributed to the pension fund, that is, they are unemployed.

The “Family Basket Bonus” is a subsidy that can be collected by mothers who are beneficiaries of the “Juana Azurduy bonus” and people with disabilities, including the blind and people with “moderate disabilities”. There are around 90,000 disabled people and 250,000 mothers who are qualifying in this condition to receive a subsidy.

The “Family Bonus” is the one that is granted to the most vulnerable families. If a worker has already received the “Universal Bonus” they can no longer receive the “Family Bonus”. Despite the low amounts of subsidies, social programs in Bolivia have made it possible to significantly reduce poverty and inequality. Thus, Bolivia has managed to go from a poverty level of 59.9% in 2006, to 36.4% by the end of 2017, a number that represents the lowest historical level in Bolivia. Regarding income inequality measured by the Gini index, it has been reduced from 0.60 to 0.47 between the years 2015–2014 [2].

Among the various social programs in Bolivia, we have “Bono Juancito Pinto” which was the first of the three social support programs that Bolivia has. It is a program that began in October 2006. Its objective is to encourage enrollment, permanence and completion of the school year for boys and girls during primary school. The program provides an annual bonus of 200 bolivianos (approximately US$28) to public school students on the condition that they maintain attendance of at least 80%.

According to data made public by the Bolivian Ministry of Education, in 2018, 444 million Bolivians were allocated for the payment of the Bono Juancito Pinto, and it will benefit some 2,221,000 primary and secondary students from the different public schools. Likewise, the "Juancito Pinto" Bonus has reduced school dropout from 6.5% to 1.8%, while for the same period in secondary education the reduction in dropout has fallen from 8.5% to 4%.

The second of three programs that was launched was the “Renta Dignidad”, which came into force in November 2007. The “Renta Dignidad” is a non-contributory monetary transfer program for life for people over 60 years of age. or more. It is a complement of 250 bolivianos (US$ 36.22 US dollars) for retired people and with social security income. In addition, a subsidy of 300 bolivianos (US$46.43 US dollars) is granted for people who do not have access to a social security income. This subsidy is financed through the Direct Tax on Hydrocarbons (IDH) and dividends from public companies. Subsidy coverage reaches 12% of the population.

The third social program is the "Juana Azurduy" and which is linked to the Covid 19 Bonus for Bolivia. The “Juana Azurduy” program is a conditional cash transfer mechanism implemented by the Bolivian Ministry of Health since 2009. It is an economic incentive for pregnant women conditional on pre- and post-natal control. The subsidy can be extended until the child is two years old. The financing of the "Juana Azurduy" bond is made through the internal resources of the General Treasury of the Bolivian Nation. The coverage of this program reaches about two million people.

### 2.6 Colombian case [See 20]

In Colombia, only a solidarity bonus equivalent to 120 dollars would have been granted, which would imply that together with Bolivia they are the countries that have the least resources for social activities. The program has reached a coverage of 30 million Colombians. The distribution of the money was made in three deliveries of 160,000 peos (US$ 40 dollars).

The Colombian citizen enters the link: https://ingresosolidario.dnp.gov.co/ and verifies if he is entitled to the solidarity bonus. The procedure indicates in which bank the respective collection will be made, through the following banks:

- Bancolombia that covers 51 municipalities and served 160,000 beneficiaries.
- Bancamía that covers 95 municipalities and served 123,000 beneficiaries.
- Banco Caja Social that covers 22 municipalities and paid 51,000 beneficiaries.
- Movii with participation in 10 municipalities for 18,000 beneficiary households

In the event that the beneficiary did not receive the delivery, all will be made in a single transfer, through the National Planning Directorate (DNP) of Colombia [21]. Vulnerable households that are not part of the Familias en Acción, Jovenes en Acción, Colombia Mayor and VAT compensation programs are participants.

### 2.7 Comparative descriptive analysis of countries

Both in the Latin American region and in different regions of the world, a series of subsidies have been granted to offset drop in consumption of vulnerable families [22].

The figure below shows the amount of the pandemic bonus (in US dollars). Peru is one of the countries that would grant an average bonus per person within the average of the Latin American region.

Fig 1 below, shows that US leads the average delivery of Bonds per person. Peru achieved average bonus per person in Latin America. Many of the allocations granted to the countries in the figure have been monthly or have covered different segments of the population.

**Fig 2:**
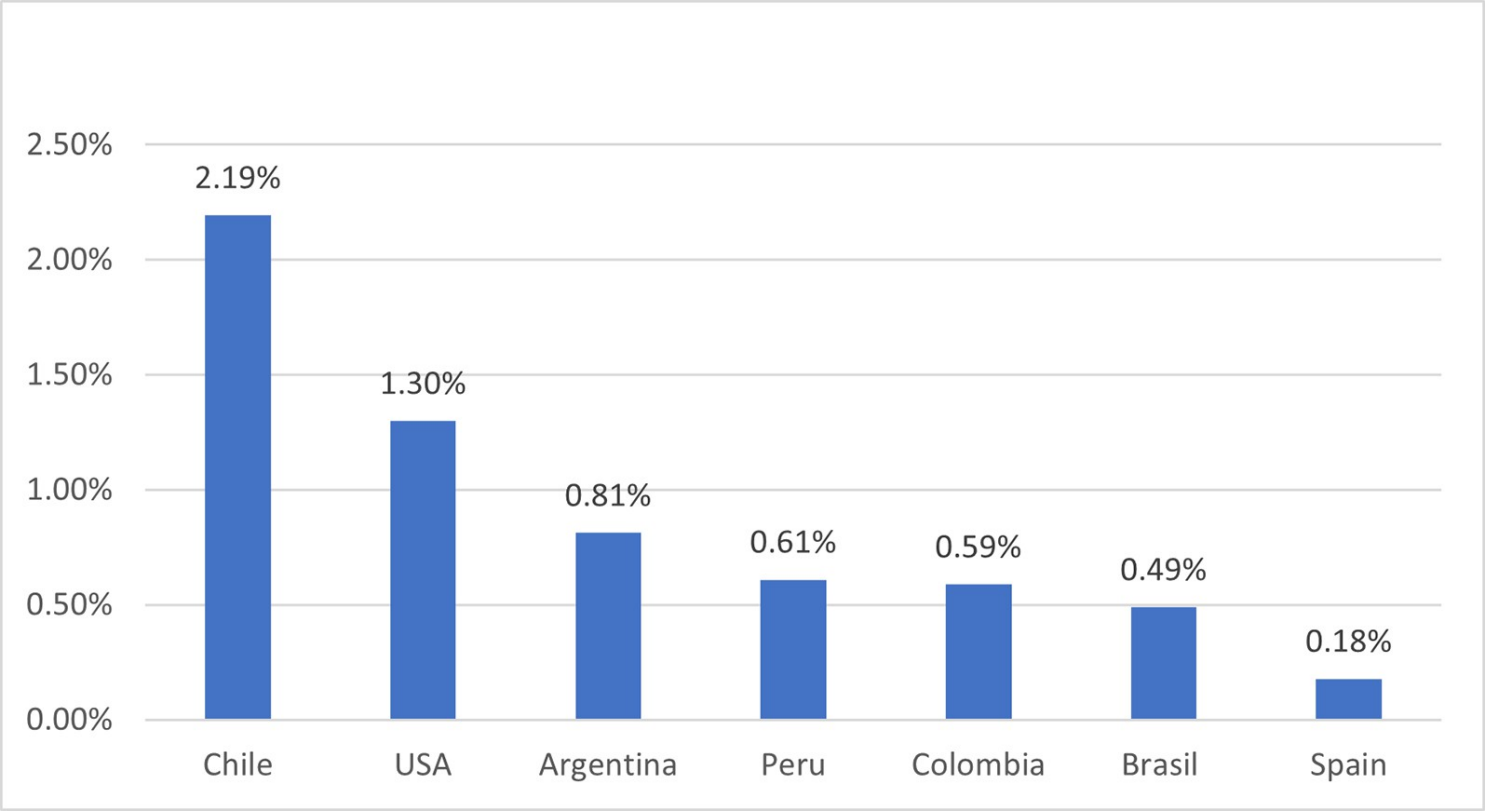
COVID monetary subsidy (%de PBI). **Source:** Central Banks [15].

Fig 2, below shows the latter subsidy as shares of GDP. Peru is still within the average in the provision of demand stimulus. In the following sections of the paper, we will analyze the bonus granting mechanisms for some countries in the Latin American region. The latter Case Study, will permit us to explore in depth the reasons for the heterogeneity of economic and health result after the policy of financial support.

The subsidy policies mentioned in the section were set to avoid COVID contagious due to the lockdown of the economy. Next section will analyze the effect of subsidies in Peru. We do not have data to make comparison of several countries in Latin-American Region. Peru is relevant to study since it had a large number of COVID deaths and the economy drops significantly in 2020. (World Bank Reports, 2020).

### 3. Difference and Difference Model (DID) for the assessment of the subsidy to the poor

In this section, the difference regression technique will be used to determine the effect on health of vulnerable families before the granting of State bonds in times of Pandemic.

The Regressions in Difference Technique or Differences in Differences (DID) is a quasi-experimental technique widely used in econometrics. This technique measures the effect of a treatment in a given period of time. The technique is used to measure the change induced through a particular treatment or event. It consists of measuring the effect that an intervention has on a treatment group with respect to a comparison group that does not have the intervention over time. It is widely used in medicine to verify treatment of a certain medicine and also in "Cash Transfer" programs or money transfer to vulnerable populations [23, 24]. For example, there are studies in Latin America on the effect of the pro-youth program and in Argentina there are studies. The technique allows establishing causality of a treatment or program in populations treated over time.

The term “difference-in-differences (DID)” calculates the effect of the intervention as a difference between two differences. The first subtraction comes from what is observed in the treatment group after receiving the bonus or program minus the result that is verified before receiving the bonus or program; and the second subtraction would be the result that is observed in the comparison group after the voucher or program minus the result that was observed before (without any intervention). Thus, to calculate the difference in differences, the first subtraction is subtracted from the second subtraction. In this way, DD calculates how much the group that received the bonus changed over time compared to how much the comparison group (which received nothing) changed over time.

The typical DID model is:

$$Y_{it=}\gamma_{s(i)}+\lambda_{t}+ðI(\ldots )+{ɛ}_{it}$$ (1)

Where *Y_it_* is the dependant variable for individual “i” and time “t”. The variable s(i) defines a group where i belongs (i.e. Treatment or Control group). I() permits to shape the event of treatment with a dummy condition (specified by …). The coefficient “ð” measures the treatment effect.

All the assumptions of the OLS model apply equally to DID. In addition, DID requires a parallel trend assumption. The parallel trend assumption says that: λ1 = λ2 are the same in both S = 1 and S = 2.

**Fig 3:**
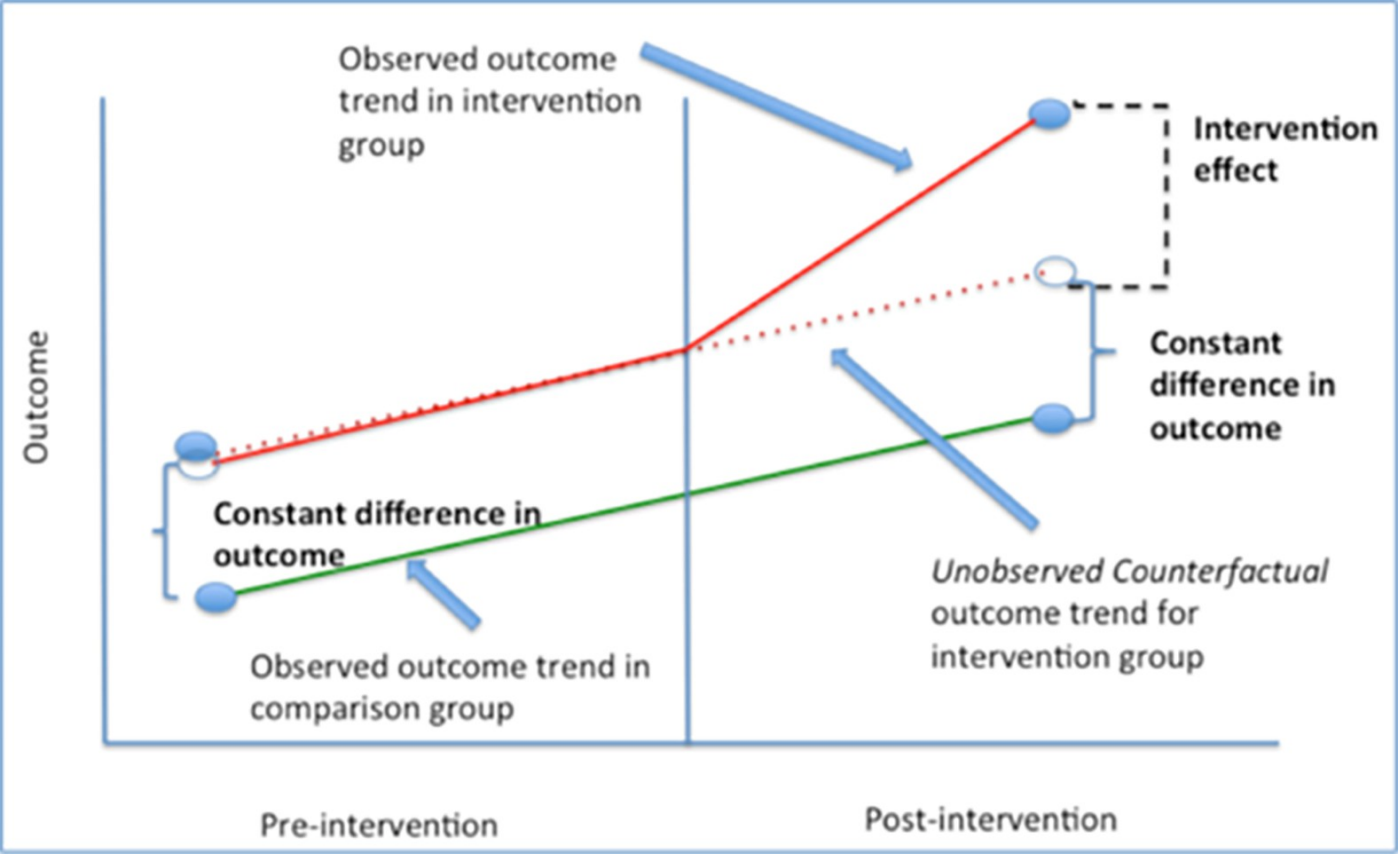
DID graphical explanation. **Source**: See [26].

The main idea of the Model can be summarized in the following Fig 3 (See [25] for more details about the technique).

As we can see from Fig 3 above. There are two stages considered in the DID assessment: before and after treatment. The two groups may have different final outcomes over time with or without treatment. The effect as it is show in the figure above, can be constant or with a trend. This technique comes from medicine, but it is very popular in economics as well. Next section will describe the data and the DID model application to respond our hypothesis.

[27] shows a concern with DD models which is that the program and intervention groups may differ in ways that would affect their trends over time, or their compositions may change over time. Propensity score methods (PSM) are commonly used to handle this type of confounding in other non-experimental studies. In our case the nature of the subsidy program for vulnerable does not differ over a short period of time under consideration. The subsidy is applied within a month massively so that there is not need to consider a more complete technique to test the effectiveness of the COVID subsidy policy

## 4. Data and empirical DID model

We have DATA from the Instituto Nacional de Estadística e Informática (INEI), internal data from the Peruvian Health Sector as well as socioeconomic variables of vulnerable (Internal Survey used to provide the financial support). The information provided by INEI contains income, socioeconomic and mobilization of the economic agents across Lima and Peru. We also used Data from University of Maryland regarding mobilization during the Pandemic. Data for Health is gathered from MINSA. The period under assessment starts in October 2019, May and June 2020.

Therefore we have a Panel with respondents across three periods of time. The first period of time is before COVID Pandemic and the next two periods are in the middle of the first COVID wave. In the Data, we can identify two different groups that are similar each other (vulnerable and qualify for subsidies described above). In the second period, one group receives the subsidy (treatment) and other does not receive the aid (control). The last period is a period of time where the health situation worsens.

We are able to perform DID analysis since the Data contains two groups: control (without bonus and poor) and treated (with bonus and poor) over three different periods of time. The periods of time under assessment are relevant to study the cost benefit of the subsidies to the poor during the Pandemic.

The Subsidy “Yo me Quedo en Casa” was the first to be provided for poor assistante and offset drop in consumption of vulnerable population. Also, it as the name claims, it is supposed to make the poor to stay at home with the subsidy and avoid working outside and get contagious by COVID disease. We may expect that the number of cases will drop as well as the economy will not be dampened. We are able to compare the group of “Yo me quedo en Casa” against other groups that did not received the Bonus over the period of time under assessment. The control group are not in the “Yo me quedo en Casa” program but they may either receive other type of Bonds or nothing (Sources from the media). The other programs came after some periods of time out of our range of assessment. Then we are able to split the sample in control and treatment groups. According to Emergency Decrees 27 and 44, “Yo me quedo en caso” bond was delivered in April 2020. The time dummie starts in may 2020.

Our empirical model will consider the following specification:

$${DCOVID}_{it=}\gamma_{s(i)}+\lambda_{t}+ðI(Bond)+{ɛ}_{it}$$

The variable “DCOVID” is a dummy that takes the value of 1 if an individual “i” die of COVID at time t”. The dummie “I” is set to be one if an individual receives a Bond and zero otherwise. There are two groups (S_i_ = 1,2) in the sample and t = 1,2 and 3. The treatment effect is captured in “ð”. The variable λ_*t*_ captures trend over time and *γ_s(i)_* considers trend by groups.

### 4.1 Results of the DID model

Table 2 shows statistical summary of variables that will be used in the DID model. We have plenty of data to perform an inference.

**Table 2:** Summary statistics.

Variable	Observations	Mean	Std Dev	Min	Max
Gender	208614	0.785	0.24	0	1
Age	80598	29.33	9.43	0	50
Treated Dummie	208614	0.51	0.5	0	1
COVID Death Dummie	208614	0.06	0.25	0	1
Income	208614	1375.5	1789.6	0	179079

The following Table 3 shows the results for the DID model. We are able to differentiate the treatment and control groups regarding health results.

**Table 3:** DID model results: Testing cost benefit of subsidies to vulnerable families.

**Observations**		238,914		
**Groups**	**Before**	**After**	***Total***	
Control	21,546	120,345	141,891	
Treatment*	24,678	72,345	97,023	
***Total***	46,224	192,690	238,914	
**Variable**	Deaths by Covid	Standard Deviation	Value |t|	p-value
*Before*				
Control	5.56			
Treatment*	5.47			
**Diference** (T-C)	-0.09	0.005	12.76	0.00***
*After*				
Control	5.52			
Treatment*	5.40			
**Diference** (T-C)	-0.12	0.003	71.25	0.00***
*Difference in Difference*	-0.003	0.006	0.17	0.812

R^2^: 0,03. Level of Confidence:

*** 99% (p<0.01)

** 95% (p<0.05)

* 90% (p<0.10)

Considering whether each individual died from COVID and differentiating by treatment groups, it can be seen that there is no significant difference (in terms of deaths from COVID) between the groups that received treatment and those that did not. In other words, receiving the bonus or not did not necessarily lead to a reduction deaths for COVID among the beneficiaries of the bonus. This result is in line with what was found above in the Case study: relative low provision of resources assigned to the poor during the Emergency State was not enough to lockdown completely the poor to avoid contagion and consequently, drop the number of deaths by COVID: There is not health benefit from providing subsidies to the poor and the cost was about 0.6% of Peruvian GDP [28]. See [3, 29] for further details. The minimum wage in Peru is US$ 300 dollars and the basket of consumption for the vulnerable is US$ 100 dollars a month. The amount given for a program was not enough and the people mobilize at the risk of COVID contagious. Our period of time under assessment considers one program that applies for a single vulnerable.

**Fig 4:**
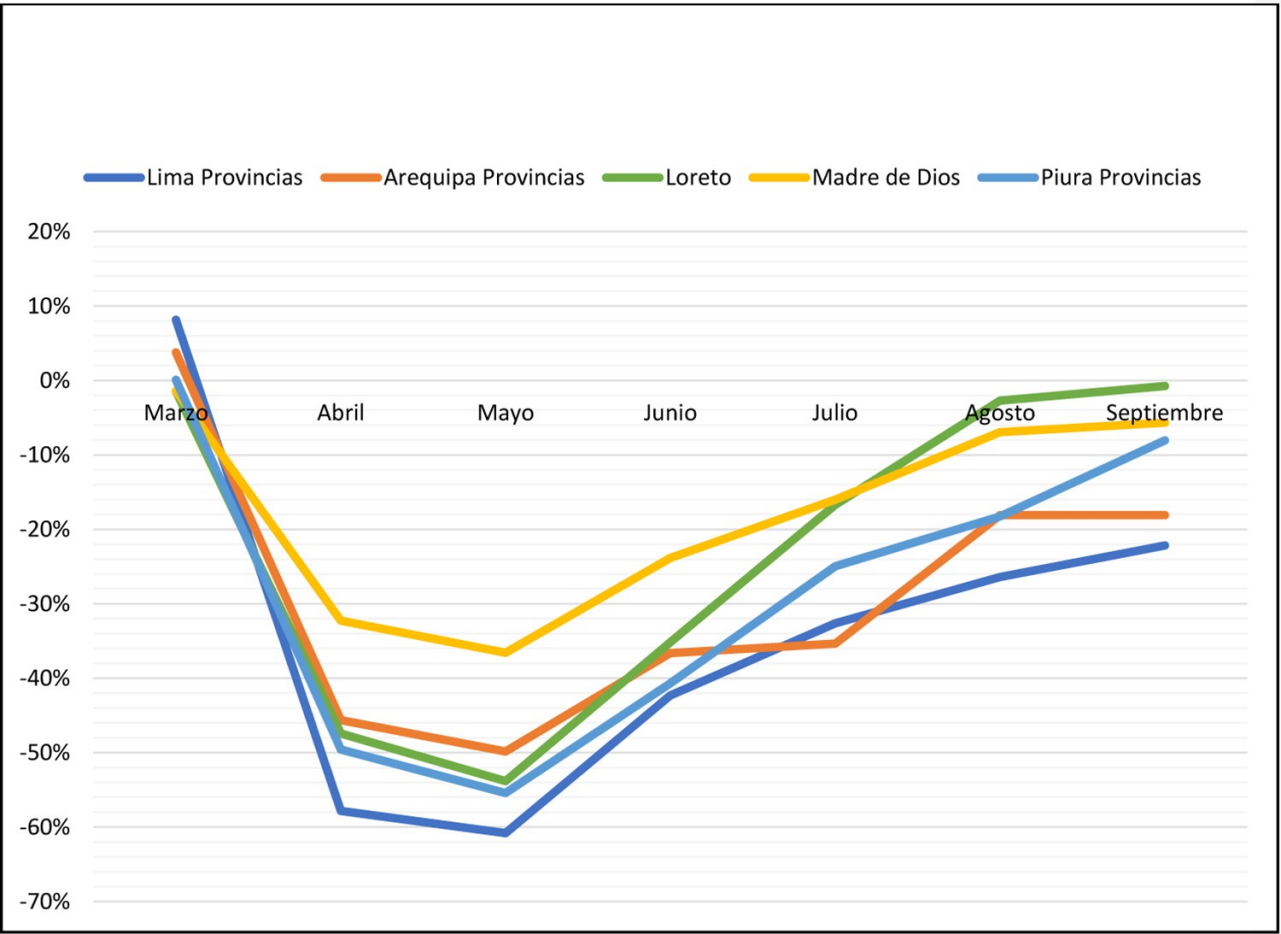
Maryland’s index mobilization in Peru (Base February 2020). **Source:** Maryland University [30].

We can see from Fig 4 below that despite the provision of social assistance during pandemic, people mobilize. The figure shows an indicator of mobilization coming from the data of the University of Maryland. The indicator is constructed according to latitudes provided by cell phones. If the mobilization drops, the people stay at home otherwise the people move. We can see from figure that after a short period of time, the people in average move. [28] got heterogeneous results of mobilization for the US

Therefore, the Bond does not hit the main purpose to lockdown ([31] got same results for a sample of African American. However, [32] obtained a positive result in the financial assistance for farm operations in the US.) the household, reducing probability to get COVID and stay safe. [31] got same results for a sample of African American. However, [32] obtained a positive result in the financial assistance for farm operations in the US. In addition, the historical drop in the Peruvian economy is another indicator that shows the failure in the provision of social assistance to the poor during the pandemic.

## 5. Conclusions

It has been verified that the disbursement granted by the government has been below the average of the region. The disbursement has been granted only once in a period of austerity and quarantine of almost six months. This situation generated movement of agents, increasing the probability of contagion and therefore death from COVID.

Then we have verified in the DID model that there is no significant difference between the treatment and control groups in terms of deaths. We may infer the latter conclusion since the treatment variable effect resulted with a non-significant sign.

For policy implications, our study helps the policymaker to understand the difference between the subsidy policy applied and therefore, contrast policy results expected vs realized impact. Since we suggest that there is no significant difference in COVID related deaths comparing those who received the funds and those who did not, individuals mobilize because of the lack of effective subsidy policy. Our research permit us to learn from this experience and applied a more effective policy for a next Pandemic episode.

## Supporting information

S1 Data (ZIP)

S2 Data (ZIP)
